# Flexible perylenediimide/GaN organic–inorganic hybrid system with exciting optical and interfacial properties

**DOI:** 10.1038/s41598-020-67531-3

**Published:** 2020-06-26

**Authors:** Rachana Kumar, Sunil Singh Kushvaha, Mahesh Kumar, Muthusamy Senthil Kumar, Govind Gupta, Kavindra Kandpal, Pramod Kumar

**Affiliations:** 1 Photovoltaic Metrology Group, CSIR-National Physical Laboratory, Dr. K. S. Krishnan Marg, New Delhi, 110012 India; 2 2D Physics and QHR Metrology Group, CSIR-National Physical Laboratory, Dr. K. S. Krishnan Marg, New Delhi, 110012 India; 3 Photonics Materials Metrology Group, CSIR-National Physical Laboratory, Dr. K. S. Krishnan Marg, New Delhi, 110012 India; 4 Thin Film Gas Metrology Group, CSIR-National Physical Laboratory, Dr. K. S. Krishnan Marg, New Delhi, 110012 India; 5 Department of Electronics and Communication Engineering, Indian Institute of Information Technology Allahabad, Prayagraj, 211015 India; 6 Spintronics and Magnetic Materials Laboratory, Department of Applied Sciences, Indian Institute of Information Technology Allahabad, Prayagraj, 211015 India

**Keywords:** Materials for devices, Electronic materials, Optical materials

## Abstract

We report the band gap tuning and facilitated charge transport at perylenediimide (PDI)/GaN interface in organic–inorganic hybrid nanostructure system over flexible titanium (Ti) foil. Energy levels of the materials perfectly align and facilitate high efficiency charge transfer from electron rich *n*-GaN to electron deficient PDI molecules. Proper interface formation resulted in band gap tuning as well as facilitated electron transport as evident in I–V characteristics. Growth of PDI/GaN hybrid system with band gap tuning from ultra-violet to visible region and excellent electrical properties open up new paradigm for fabrication of efficient optoelectronics devices on flexible substrates.

## Introduction

In the current era of modern technology and smart devices, the hybrid nanostructured materials are becoming integral component of devices^1–3^. Optical light emitting diodes (LED), sensors, detectors, transistors and photovoltaics have shown exciting performance with shifting paradigm from pure inorganic to organic–inorganic hybrid systems where, hybrid materials form long range interface at the molecular level^4–11^. In general organic–inorganic hybrids are composed of two or more components, typically inorganic and organic molecules, interacting together by weak physical or strong chemical bonds^12^. The physical and chemical properties of the hybrid system is exhibited not simply from the sum of individual contributions but as the synergistic reinforcement of specific properties. Originally organic–inorganic hybrid materials were reported for the design of hybrid polymers, silicon-carbon networks etc.^13–18^. However, in the recent years several semiconductor organic–inorganic hybrids have come into reports for the tuning of band gap as well as enhancement of other optical and electronic properties for versatile applications^19,20^. Excellent properties of MXene has been exploited by preparation of MXene-contacted Si solar cells delivering high power conversion efficiency due to improved interfacial properties^21^. Self-powered wearable photodetectors have been developed using composite of ZnO:polyvinylidene fluoride^22^ and solid state polyaniline:MgZnO bilayer^23^.

Gallium nitride (GaN) is a very promising semiconductor for hybrid optoelectronic devices due to its high exciton energy, wide band gap and high mobility^24,25^. Organic–inorganic interface has been tailored by applying different organic materials over GaN to obtain desired transport and optical properties. Pt nanoparticles coated GaN nanowires based field emitters showed reduced turn on voltage compared to neat GaN nanowires^26^. The better performance was attributed to the electron states distribution near Fermi level providing more electrons to the conduction band at applied operating voltage. Moreover, integration of GaN with organic materials has been studied for generation of white light mostly on rigid substrates^27–30^. Organic/GaN hybrids are of special interest to fabricate high performance power devices. Several polymer based materials have been used for down conversion of emission radiations^31^. The efficiency of colour conversion is greatly enhanced due to nonradiative Foster resonance energy transfer (FRET) between GaN and organic molecules. Reports are available on functionalization of GaN with organic molecules/biomolecules and have opened up application of III-nitride wide band gap systems in new fields of applications^32,33^. Most of the studies have been performed using electron rich (p-type) organic semiconductor materials over GaN like PEDOT:PSS, F8BT, MEH-PPV, porphyrin, graphene etc.^34^. Such hybrid materials take the advantage of special optical properties of organic semiconductor in combination with excellent electrical properties of inorganic semiconductor. There are only a few studies on GaN based hybrid systems using electron deficient (n-type) organic materials. Schultz et al., demonstrated the tuning of GaN work function from 4.2 to 6.0 eV by using organic acceptors^35^. Wang et al. demonstrated tuning of work function of H-Si(111) using two cyano-quinodimethane derivatives^36^. Wang et al., have studied the molecular alignment and electronic structure of thermally deposited perylenetetracarboxylic dianhydride (PTCDA) molecules over black phosphorous^37^. PTCDA has also been deposited over gold Au(111) and studied for their thermoelectric properties^38^. Thin interlayer of PTCDA at Ag/n-GaAs contact decreases the Schottky barrier height from 0.81 to 0.64 eV^39^. So far only a few organic acceptors are reported^40^ for preparation of organic/GaN hybrid nanostructured materials for tuning of optical/electrical properties and require more research in this area.

**Figure 1:**
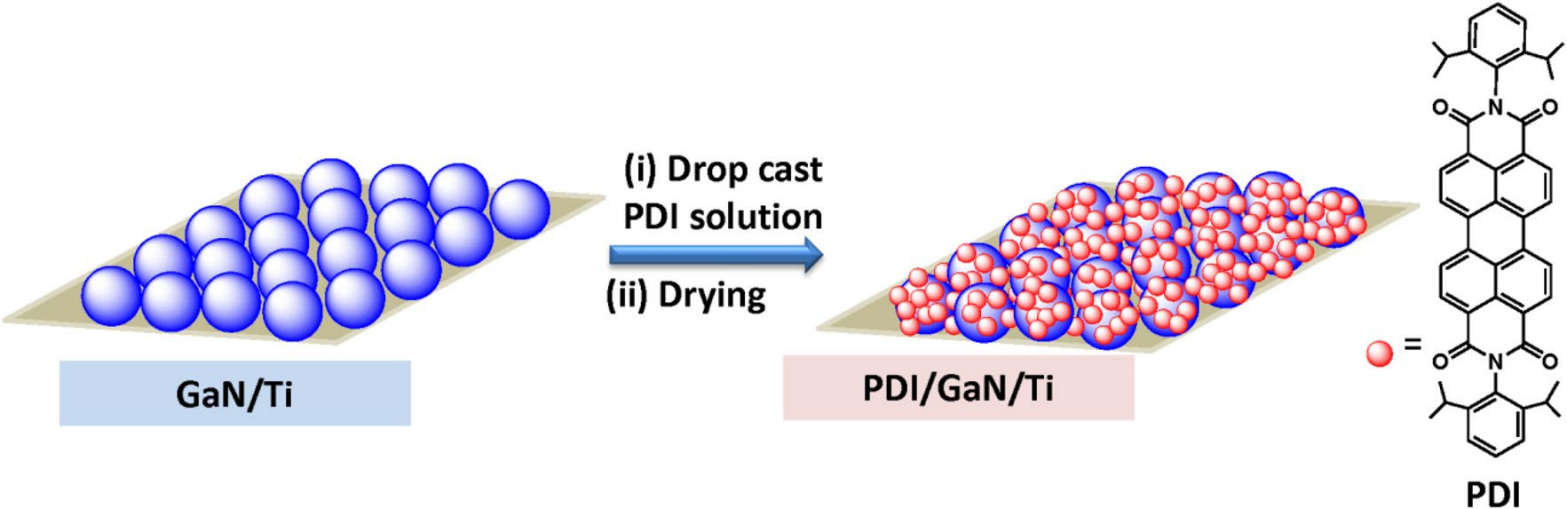
Schematic for preparation of PDI/GaN/Ti hybrid system.

The present work comprises preparation of perylenediimide (PDI)/GaN hybrid system by drop casting PDI solution on laser molecular beam epitaxy (LMBE) grown undoped n-GaN film over flexible titanium (Ti) metal foil (Fig. 1)^41^. PDI is electron deficient organic semiconductor material possessing highly applicable properties like, strong light absorption in the range of 400–700 nm due to their extended conjugated-π systems^42^, self assembling properties^43–46^, excellent electron mobility^47^ etc.^48–50^. To synergize the advantages of GaN and PDI, the hybrid of PDI over GaN has been fabricated to deliver exciting optical and interfacial properties. Absorption, photoluminescence (PL), time resolved photoluminescence (TRPL) and transient absorption spectroscopy (TAS) have been employed to understand the energy level tuning and charge transfer interactions between PDI and GaN molecules. The dispersion of PDI molecules over GaN surface has been probed by different microscopy study. Modification of Pt/GaN Schottky contact in hybrid is investigated using current (I)–voltage (V) measurement by conducting atomic force microscopy (c-AFM). A thorough description of interaction between PDI and GaN molecules with its implication on optical and interfacial electrical properties has been given.

## Results and discussion

The ratio of Ga to N plays a critical role in determining the GaN growth modes in III-nitride MBE process. Two dimensional (2D) GaN growth mode occurs under Ga-rich condition whereas, N-rich condition promotes 3D GaN growth^51^. In the current work, GaN growth was performed under N-rich condition as additional nitrogen species was supplied during ablation of stoichiometric solid GaN target^41,51^. The Ga and GaN_1−x_ ad-atom surface diffusion is low on polycrystalline Ti surface at growth temperature of 600 °C favouring the growth of GaN islands on Ti foil under N-rich growth condition. On the other hand, PDIs are highly fluorescing dyes in their J-aggregates however; J-aggregation results in poor electrical conductivity. PDIs with H-aggregation behaviour show high electrical conductivity but suffer with fluorescence quenching due to nonradiative energy transfer in aggregated state. We have reported PDI derivatives showing excellent fluorescence with high electrical conductivity due to slipped π–π stacking^52,53^. In the current work we used 2,6 diisopropyl phenyl substituted PDI (*i*PrP-PDI, will be referred as PDI in rest of the manuscript, Fig. 1) with disturbed π–π stacking resulting in high solubility with decent electrical conductivity and electron mobility^52^. The HOMO–LUMO energy levels were calculated to be ~ − 5.9 and ~ − 3.8 eV respectively and suitably match with GaN energy levels. The hybrid system was fabricated as shown in Fig. 1 by drop casting the chloroform solution of PDI over GaN/Ti followed by annealing to remove the solvent and attain homogeneous dispersion.

**Figure 2:**
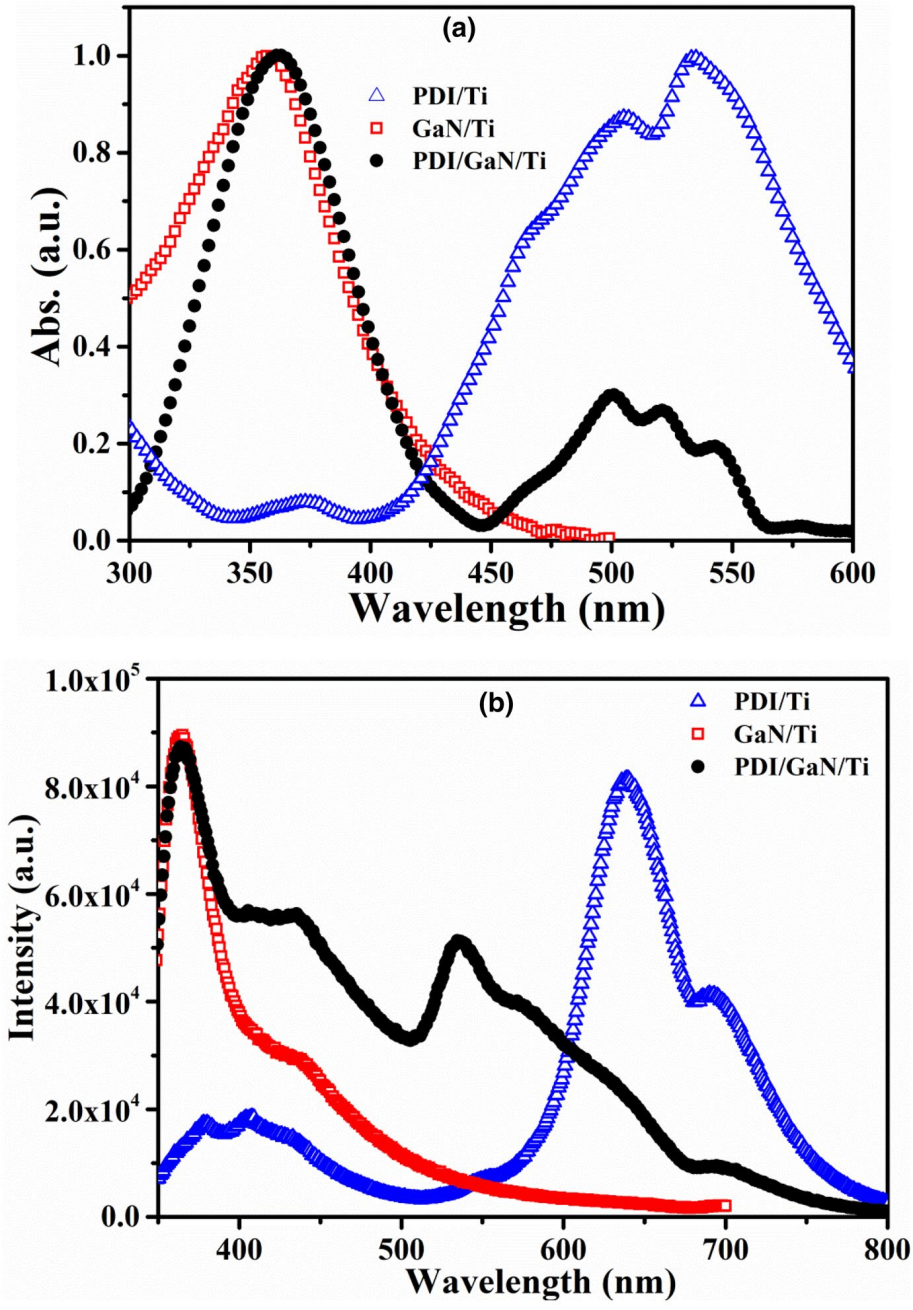
(**a**) Normalized absorption and (**b**) photoluminescence spectra of GaN/Ti, PDI/Ti and PDI/GaN/Ti.

To evaluate the optical properties of GaN/Ti and PDI/GaN/Ti hybrid nanostructure system, absorption and PL spectroscopy studies were performed. The absorption spectra of GaN/Ti, PDI/Ti and PDI/GaN/Ti are shown in Fig. 2a. GaN/Ti shows absorption at 358 nm equivalent to its direct band gap energy (~ 3.46 eV). PDI/Ti shows absorption bands at 507 and 532 nm for 0–1 and 0–0 π–π* electronic transitions, respectively. The absorption bands observed are similar to PDI films over glass substrates^52^. PDI/GaN/Ti shows absorption bands at 361, 501, 521, 543 and 578 nm where, GaN absorption band is red shifted while the PDI absorption bands are blue shifted (owing to H-type aggregate formation). There is also appearance of new absorption bands at 543 and 578 nm in hybrid for charge transfer (CT) states as also seen with organic donor and PDI nanostructured co-assembly^54,55^. The appearance of CT bands suggests ground state interaction between the organic and inorganic semiconductors resulting in tuning of band gap in hybrid nanostructure. The formation of CT excitons can be exploited for photovoltaic and photodiode properties. To get further insight into CT between PDI and GaN, we performed PL and TRPL study.

Figure 2b represents the PL spectra of GaN/Ti, PDI/Ti and PDI/GaN/Ti hybrid structure using 325 nm excitation wavelength at room temperature. GaN/Ti emits at ~ 3.41 eV (364 nm) for near band edge emission of excitonic states with full width at half maximum (FWHM) of ~ 260 meV^56^. These values confirm that wurtzite GaN films are grown over Ti foil and it can be potentially used for hybrid optoelectronic device fabrications. PDI/Ti shows bands at 639 nm for 0–0 transition and 691 nm for 1–0 transition which are highly red shifted compared to PDI films^52^ but similar to what observed for perylenediimide microwires for effective wave guiding due to strong π–π stacking^57^. The PDI/GaN hybrid system shows lower emission intensity at 364 nm for GaN band edge emission, 536 nm for 0–0 transition, 576 nm for 1–0 transition and 629 nm for 2–0 transition of PDI molecules. One additional emission is observed at 694 nm similar to PDI/Ti. The blue shifted emission bands in hybrid system compared to PDI/Ti are similar to observed for monomeric behaviour of PDI in solution^52^. This observation indicates towards non-aggregated dispersion of PDI molecules over GaN film forming proper interface as also ascertained by SEM analysis (discussed later).

**Figure 3:**
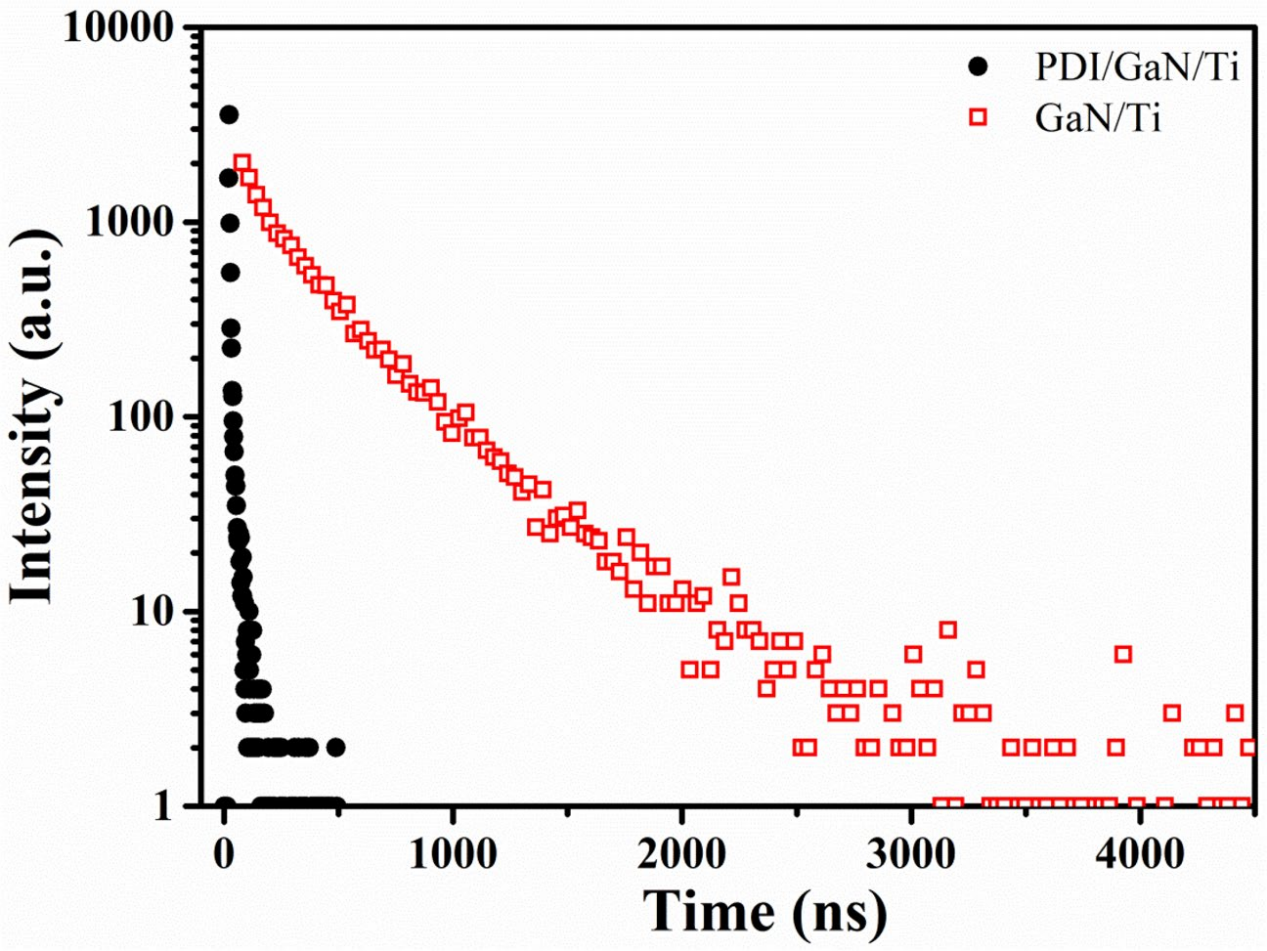
TRPL of GaN/Ti and PDI/GaN/Ti recoded at 365 nm emission at room temperature.

To find out the carrier dynamics and charge transfer in hybrid nanostructure system, TRPL was performed on GaN/Ti and PDI/GaN/Ti at room temperature. Figure 3 demonstrates the decay profile of 365 nm emission for GaN/Ti and hybrid samples. The decay was fit with two exponential function model where, A_1_ ≫ A_2_ for both the samples and therefore, the slow decay component τ_2_ is neglected^58,59^. At room temperature GaN/Ti shows the exciton lifetime of ~ 4.5 ns and decay rate is calculate to be 0.22 ns^−1^. For hybrid system the decay lifetime is highly reduced to 1 ns (~ 78% faster) with decay rate constant of 1 ns^−1^. The highly reduced lifetime is attributed towards charge transfer exciton formation at the PDI/GaN interface. The rate constant for charge transfer (k_(CT)_) is calculated to be 0.78 ns^−1^ using following Eq. 1^60^.

1 $$k_{{\left( {CT} \right)}} = \frac{1}{{\tau \left( {hybrid} \right)}} - \frac{1}{{\tau \left( {GaN} \right)}}$$

2 $$\emptyset_{{\left( {CT} \right)}} = \frac{{\frac{1}{{\tau \left( {hybrid} \right)}} - \frac{1}{{\tau \left( {GaN} \right)}}}}{{\frac{1}{{\tau \left( {hybrid} \right)}}}}$$

The efficiency of charge transfer (*ϕ*_CT_) is calculated using Eq. 2 and found to be ~ 78%. Smith et al., also observed three times higher energy transfer rate for organic/GaN/InGaN hybrid system compared to bare GaN/InGaN nanorod structure due to dominant nonradiative resonant energy transfer (RET) rate in hybrid system minimizing the nonradiative recombination losses^61^.

**Figure 4:**
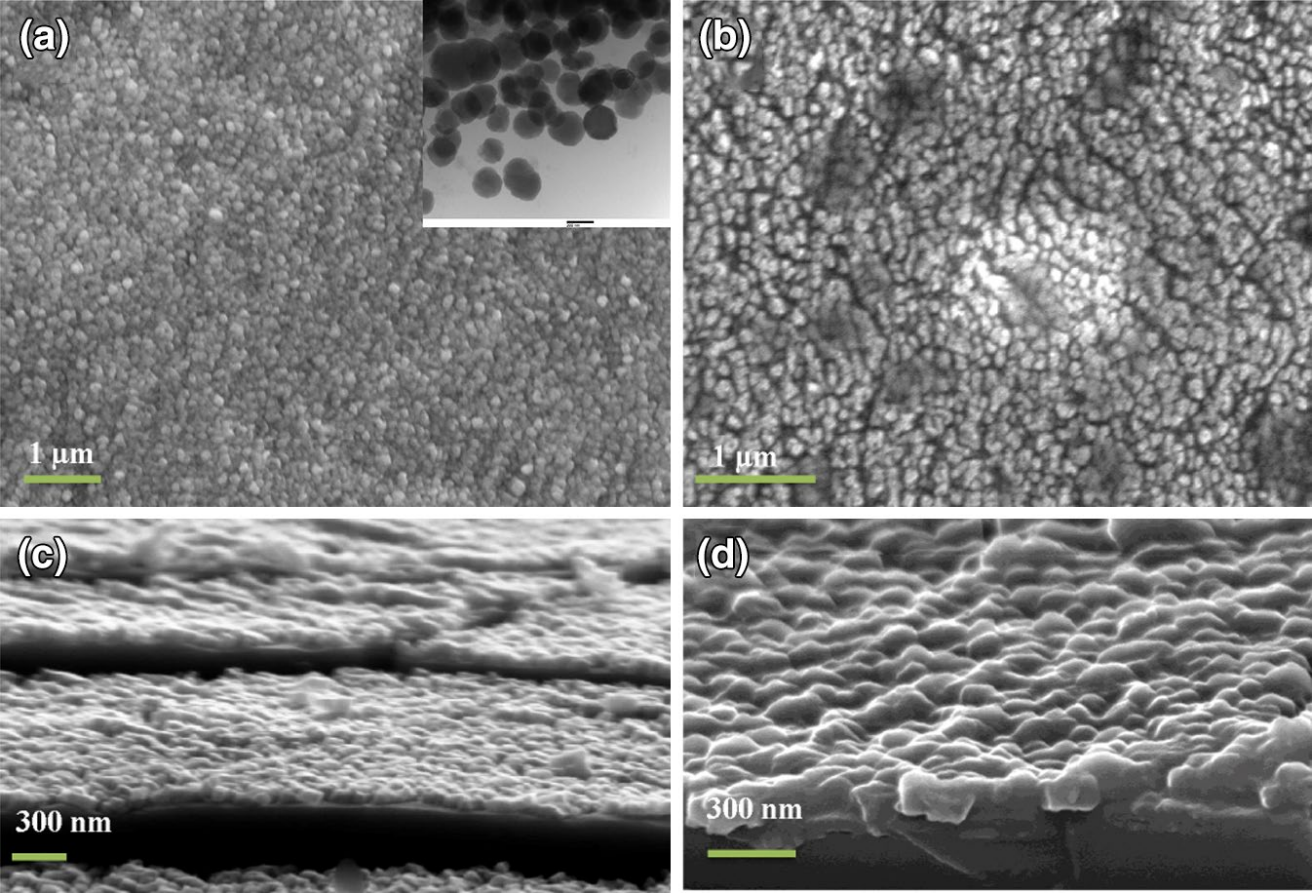
SEM images showing (**a**, **b**) surface morphology and (**c**, **d**) cross sectional view of GaN/Ti (**a**, **c**) and PDI/GaN/Ti hybrid (**b**, **d**). Inset of image (**a**) shows the TEM image of GaN where, the scale measures 200 nm.

**Figure 5:**
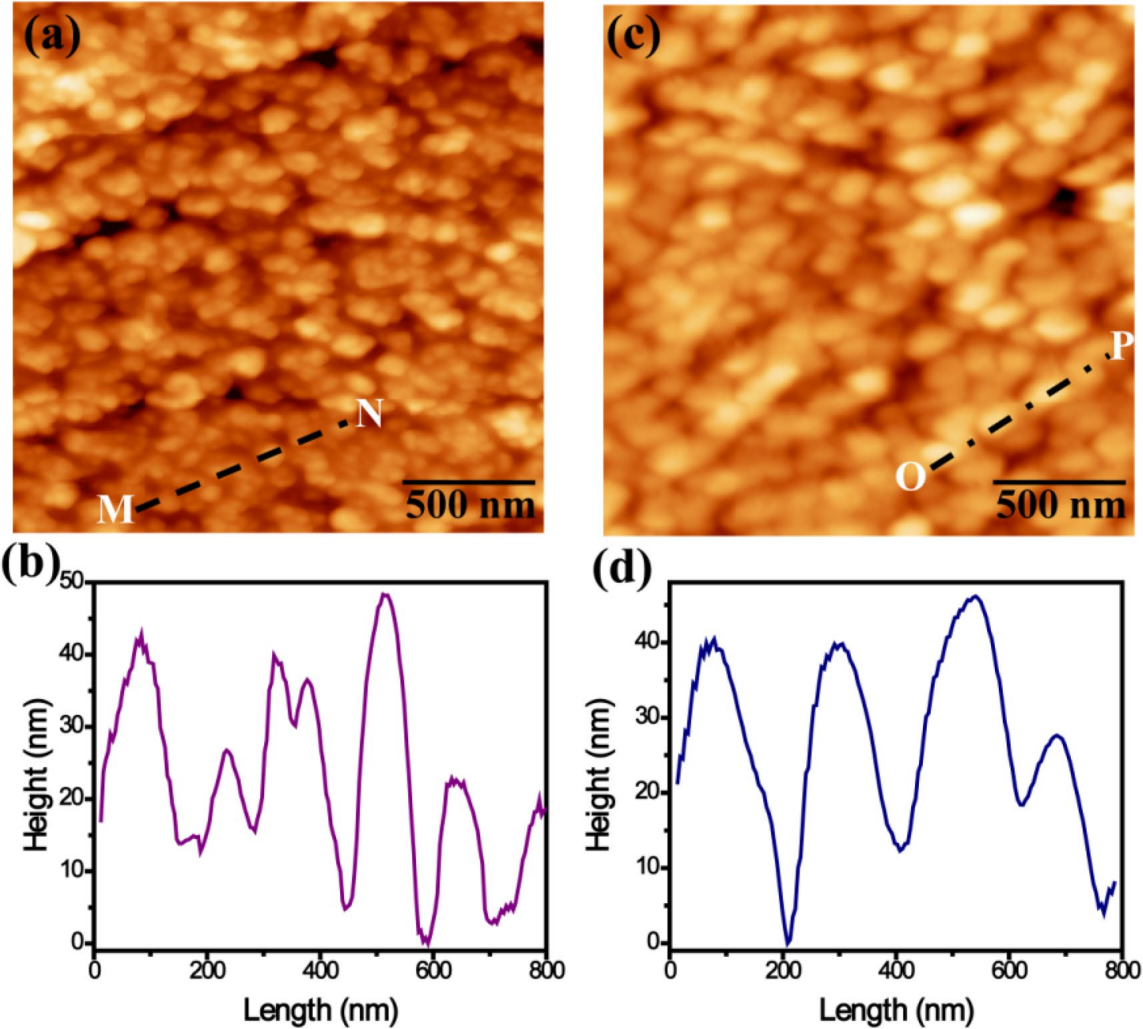
(**a**) AFM image of GaN film grown on Ti foil and (**b**) line profile across MN line in (**a**). (**c**) AFM image of PDI on GaN film grown on Ti foil and (**d**) line profile across OP line in (**c**).

Figure 4a and b shows the SEM top morphology of GaN/Ti and PDI/GaN/Ti films respectively where, GaN/Ti exhibits grain structures while it becomes more homogeneous in hybrid samples. The inset of Fig. 4a shows the transmission electron microscopy (TEM) image of GaN showing grain size in the range of 100–200 nm. Cross sectional SEM view (Fig. 4c, d) also shows uniform coating of GaN thin film with PDI molecules without aggregate formation. To further analyze the morphological properties‚ AFM was performed in tapping mode by utilizing the Si tip with radii of ~ 10 nm. The AFM image for GaN/Ti (Fig. 5a) clearly revealed the growth of granular island of GaN thin film over Ti foil. Line profile across MN line (Fig. 5b) and statistical analysis revealed the lateral size of islands in the range of 80–200 nm. Deposition of PDI over GaN/Ti surface increases the lateral size of elongated islands in the hybrid system falling between 110 and 250 nm (Fig. 5c, d). The rms surface roughness of the GaN and PDI/GaN grown over Ti foil was estimated after statistical analysis of several AFM images with scan area of 2 × 2 µm^2^ and found to be ~ 20.7 (± 2) nm and ~ 17.5 (± 1.5) nm respectively. These observations revealed that PDI molecules cover granular GaN homogenously with increase in lateral size and the proper dispersion of PDI molecules decreases the roughness of GaN/Ti in hybrid. To study the interface electrical properties of PDI/GaN/Ti hybrid system we performed the c-AFM analysis using Pt coated AFM tip in contact mode. c-AFM is widely employed to investigate the electronic properties of films.

**Figure 6:**
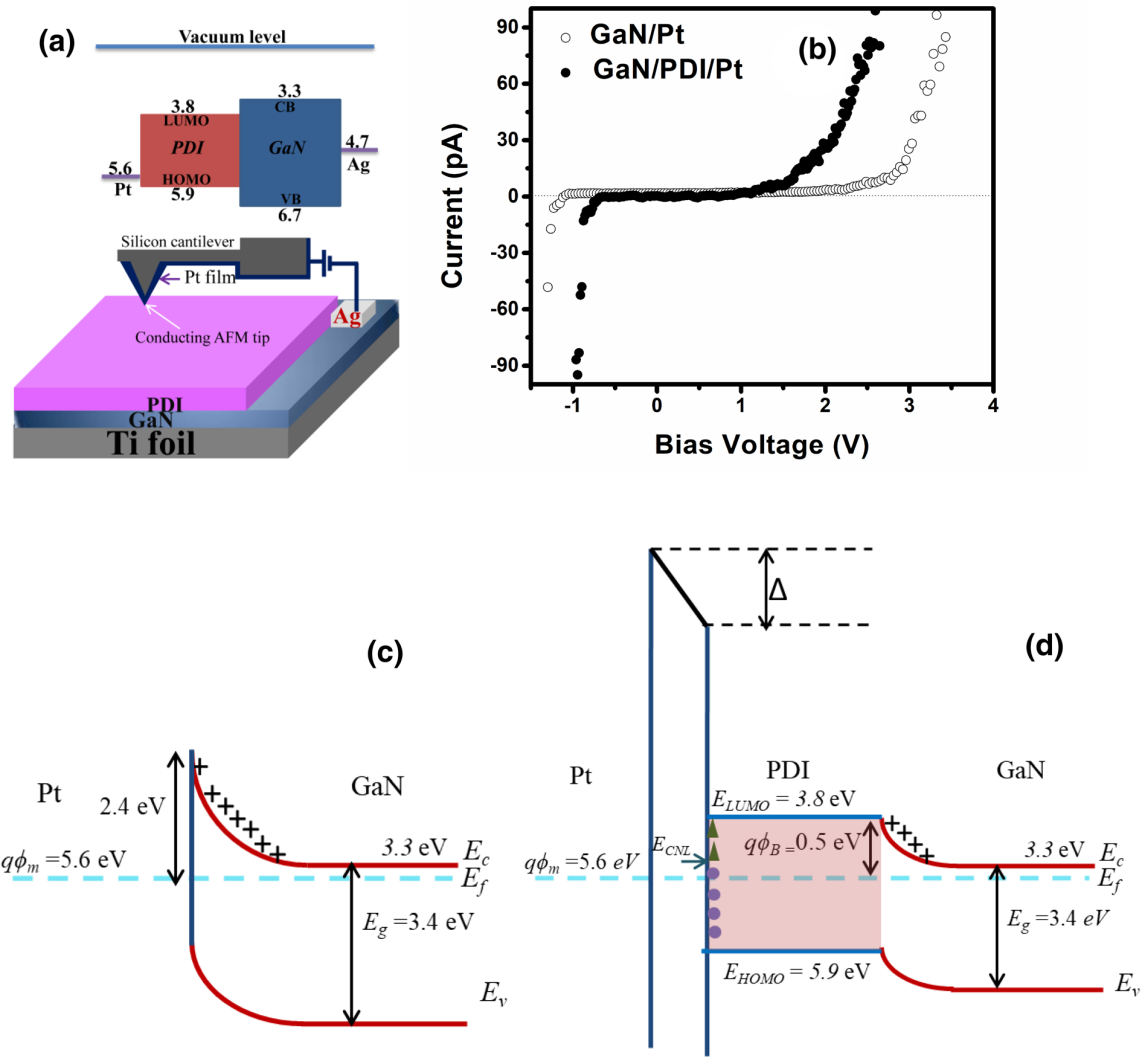
(**a**) Schematic representation of energy level diagram and I–V measurements on PDI/GaN/Ti foil using Pt coated conducting AFM tip. (**b**) I–V characteristics of bare GaN film and PDI/GaN hybrid on Ti foil. Schematic representation of energy level diagram of (**c**) GaN/Pt and (**d**) GaN/PDI/Pt Schottky junction.

Schematic of the procedure with energy level diagram is shown in Fig. 6a. The relative position of the energy levels at the interface of organic and inorganic semiconductors is an important ingredient to obtain superior properties^62^. Conducting AFM tip was kept on positive bias with respect to GaN film for hybrid system. The advantage of c-AFM measurement is related to freedom of performing I–V independently and simultaneously at various locations of the samples at nanoscale level. Figure 6b shows the experimental I–V characteristic of GaN/Pt and GaN/PDI/Pt Schottky diode. From the I–V characteristic, it is clear that the forward bias drop of GaN/Pt (1.9 V) is much higher than GaN/PDI/Pt (1.2 V). The insertion of the PDI organic layer has improved the switching characteristic of the Schottky contact^63^. Figure 6c and d shows the energy band diagram of the GaN/Pt and GaN/PDI/Pt hybrid. For GaN/Pt Shottky contact, the barrier for electron is 2.4 eV which, results in the high value of cut-in voltage. However, in GaN/PDI/Pt, due to the insertion of PDI organic material, Schottky barrier height reduces to 0.5 eV, thereby reduces the cut-in voltage. Surface states or interface dipole (0–1 nm) exists between PDI/Pt interface which accounts for Δ drop in barrier height. This interface state density introduces Fermi level pinning at the PDI/Pt interface. To explain the Fermi level pining a charge neutrality level (CNL) is defined at the interface. Above CNL level, all the surface states are considered as acceptor-like states and below CNL all the states are considered as donor-like states. The organic semiconductor PDI acts as a transport layer for the electron. As per the band-diagram‚ the GaN transfer the electrons to PDI and encounter the depletion at the PDI/GaN interface with a barrier height of 0.5 eV.

**Figure 7:**
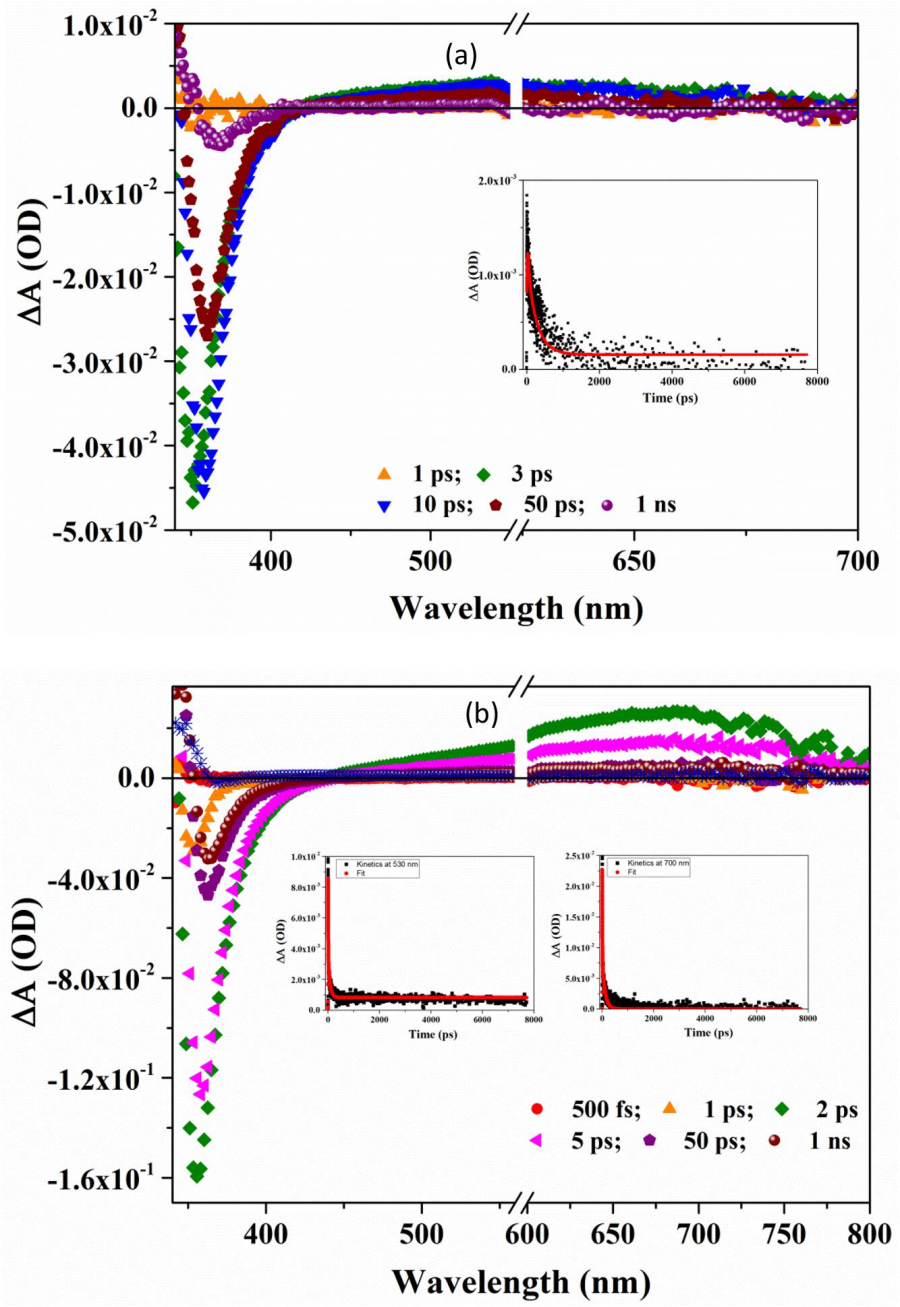
Transient absorption spectra of (**a**) GaN/Ti with decay profile at 530 nm in inset with fitting and (**b**) PDI/GaN/Ti with decay profile at 530 and 700 nm with fitting in inset using 290 nm pump wavelength. A break is shown from 560 to 600 nm for pump double resonance at 580 nm.

**Figure 8:**
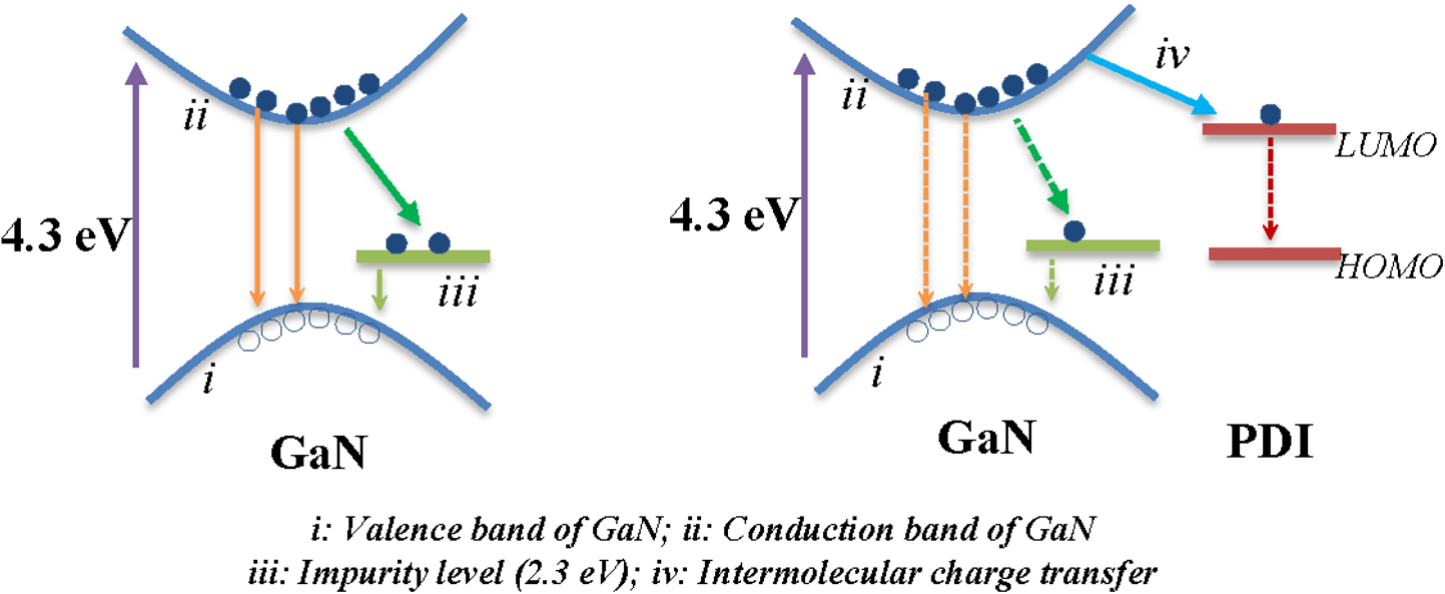
Electron transitions at the interface in GaN/Ti and PDI/GaN/Ti system in ultrafast transient absorption spectroscopy study.

Ultrafast pump-probe spectroscopy is a tool to study the photogenerated charge carrier dynamics in film as well as in solution within few hundred femto seconds of photo-excitation. A pump pulse is used with higher energy than band gap of the material for excitation and probe pulse is passed after a particular delay time to measure the differential absorption spectra. In semiconductors, the probing frequency can precisely provide information about different transitions and processes. Probing with UV–Vis range wavelength provides information about transitions associated with valence band electrons to conduction band, to the trap states or intraband transitions of electrons. The negative features (ground state bleaching, GSB) in transient absorption spectra correspond to ground state absorption (GSA), stimulated emission (SE) (decreasing population) while the positive features corresponds to photoexcited absorption (PEA) of transient species (increasing population). 290 nm (4.3 eV) pump wavelength was used for GaN/Ti to avoid resonance with exciton absorption at 360 nm. TAS spectrum of GaN/Ti (Fig. 7a) shows strong GSB at ~ 354 nm (3.50 eV) after 3 ps of photoexcitation (τ_1_ =  ~ 64 ps) and subsequently shifts to 358 (3.46 eV), 361 (3.43 eV) and 365 (3.39 eV) nm after 10 (τ_1_ =  ~ 95 ps), 50 (τ_1_ = 111 ps) and 100 ps (τ_1_ = 125 ps) respectively (parenthesis shows lifetime of signal), which may be due to exciton ionization owing to high pump energy. In GaN, free exciton composes of three bands however due to high binding energy of GaN these excitonic bands are not resolved^64,65^. At the same time after 3 ps PEA appears between 415 and 550 nm with maxima at ~ 530 nm. The band fits well with mono-exponential model function suggesting single step relaxation process with life time of ~ 85 ps (global fitting at 530 nm). The population for both GSB and PEA decreases simultaneously as ∆OD decreases with time for both the transitions and relaxes back to valence band through defects level (1.4–2.8 eV) (Fig. 8)^66^.

TAS spectra of PDI/GaN/Ti hybrid structure (Fig. 7b) also shows GSB for exciton transition with shifting of energy from 356 nm (3.48 eV) after 1 ps to 363 nm (3.41 eV) after 50 ps of photoexcitation. The lifetime is also reduced compared to GaN/Ti and varies from 1 to 50 ps. Most interestingly in hybrid nanostructure system GSB appears within 1 ps of photoexcitation while PEA appears after 1 ps of photoexcitation between 440–800 nm with maxima at ~ 700 nm. There is delay in appearance of transient species but still faster than GaN/Ti, which is basically due to intermolecular charge transfer between GaN and PDI at the interface. PDI anion radical shows the transient absorption near 700 nm in mixture with electron donor polymer poly(3-hexylthiophene) (P3HT)^52^. In the current case also we observed PDI anion radical absorption at ~ 700 nm with lifetime ~ 35 ps. The lifetime is much shorter than what was observed with P3HT:PDI solution mixtures (6 ns)^52^, however, in films the lifetimes of charge separated states are seen highly reduced^67^. The transient absorption for GaN exciton at 530 nm fits with biexponetial model function (compared to monoexponential fit in GaN/Ti) with decay time of τ_1_ ~ 5 ps and τ_2_ ~ 74 ps for PDI and GaN contribution respectively. TAS further supports the formation of charge transfer species in hybrid system as discussed above. There is effective interface formation between GaN and PDI with facilitated electron transport from electron rich GaN to electron deficient PDI. The energy level diagram for probable electron transitions is shown in Fig. 8.

On photoexcitation using the beam with higher energy than the band gap of GaN excites electron from the valence band maxima (*i*) of GaN to the conduction band (*ii*) which appears as ground state bleaching in TAS spectra. The absorption of excited electron in (*ii*) appears as transient bands (530 nm) which relaxes directly or through impurity levels (*iii*) present in material to the ground state. In PDI/GaN/Ti, the excited electrons in (*ii*) of GaN follow an additional path (i*v*) via LUMO energy level of PDI molecules forming CT states appearing as transient band at ~ 700 nm as also observed in TRPL with reduced PL life time of GaN excitons in hybrid structure.

In summary, PDI/GaN hybrid exhibits ground state charge transfer interactions and tuning of wide band gap of GaN from 3.46 to ~ 2.31 eV. PDI molecules homogeneously cover GaN thin film reducing the rms roughness to ~ 17.5 nm. Proper interface and well aligned energy level of GaN and PDI facilitates efficiency of charge transfer ~ 78% with decay rate constant of ~ 1 ns^−1^ which is as high as reported for other GaN based hybrid systems^61^. Ultra fast pump-probe spectroscopy clearly identifies the charge transfer species generated above band edge energy excitation. I–V characteristics recorded by c-AFM indicated the formation of Schottky junction at GaN to Pt and PDI/GaN to Pt interface. Due to the insertion of PDI organic material, Schottky barrier height reduced to 0.5 eV in hybrid compared to 2.4 eV barrier height at GaN/Pt. PDI is polar molecule and preferentially adsorb on defect sites of GaN film, contributing in reduction of effective barrier height. This is in line with the previous reports on Ag/PTCDA/GaAs^39^ and PEDOT:PSS/Si-GaN interfaces where organic layer preferentially adsorb on defect sites and induce reduction in barrier height explained by strong contribution of low dielectric constant of organic molecules^68^. Such properties are highly required for smart devices and PDI/GaN/Ti is envisaged as a new combination of organic n-type semiconductor-GaN hybrid nanostructured system with potential optical and electrical properties.

## Conclusions

We have established a new PDI/GaN hybrid system over flexible Ti foil and characterized using optical and electrical tools. PDI over GaN forms a proper interface and facilitates charge transfer from GaN to PDI molecules. CT band in absorption spectrum, quenched life time of GaN emission and presence of transient absorption bands for charge separated species corroborate well with the inference. PDI deposition smoothens the surface and also lower the turn on voltage in hybrid system. With this work we establish a less explored area of organic/inorganic hybrid system where an electron deficient organic semiconductor is interfaced with *n*-GaN over flexible Ti foil resulting in band gap tuning as well as exciting and promising electrical properties to be used in optoelectronic, sensor or energy harvesting devices.

## Methods

The GaN film was grown on Ti metal foil (Alpha Aesar, thickness: 0.127 mm, purity: 99.99%) using laser assisted molecular beam epitaxy (SVT Associates, Inc. USA) technique (base pressure: 2 × 10^–10^ Torr). After cleaning with organic solvents, the Ti foil was degassed in the preparation chamber for ~ 3 h at 200 °C. Later, the pre-treated Ti foil was transferred in main chamber and thermal cleaning was performed by heating at 850 °C for 30 min via infra-red radiation resistive heater. The GaN film was grown under N-rich growth condition on pre-nitridated Ti foil at 600 °C for 2 h by ablating the solid GaN target (purity: 99.9999%) via KrF excimer laser (wavelength: 248 nm) in the presence of r. f. nitrogen plasma (0.4 sccm nitrogen flow, r. f. power 250 W) and the other details are presented elsewhere^41^. *i*PrP-PDI derivative was synthesized using reported method^52^. PDI/Ti and PDI/GaN/Ti were deposited over cleaned Ti foil and GaN/Ti respectively‚ by drop casting 1 μM chloroform solution passed through 0.45 μ PTFE filter followed by annealing at 50 °C for 1 h. Absorption spectra was recorded on Avantes fiber optic spectrophotometer. The optical properties were also characterized using steady-state PL and TRPL spectroscopy using a He–Cd laser source (Melles Griot) with an excitation wavelength of 325 nm at room temperature. Zeiss EVO-MA10 scanning electron microscope (SEM) was used to analyse the surface morphology and cross-sectional view of GaN/Ti and PDI/GaN/Ti samples. The TEM sample was prepared on carbon Lacey Cu grid by dispersing the GaN and Tecnai G2 HR-TEM system was used to study the microstructure of GaN with an operating voltage of 300 kV. The Multimode-V Veeco AFM in tapping mode was performed to analyze the grain size and surface roughness whereas, I–V characteristics were measured using Pt-coated conducting AFM tip in contact mode. To study the ultrafast dynamics and charge transfer states TAS study was performed at room temperature. An optical pulse (35 fs, 4 mJ/pulse, 1 kHz, 800 nm) from Ti:Sapphire laser amplifier was split into two beams using a beam splitter. The wavelength of high intensity beam (pump) was varied from 190 to 2,600 nm by employing an optical parametric amplifier (TOPAS, Light Conversion). White light continuum (WLC) was generated from the weak intensity beam (probe) propagated through a CaF2 crystal. The probe beam was optically delayed with respect to pump beam by using a computer-controlled delay stage. A beam 290 nm pump wavelength was used at normal incidence and the change in absorption was detected by gated CMOS detector. HELIOS (Ultrafast systems) spectrometer was used for time resolved study.
